# First-Principles Calculations to Investigate the Influence of Irradiation Defects on the Swelling Behavior of Fe-13Cr Alloys

**DOI:** 10.3390/ma15031267

**Published:** 2022-02-08

**Authors:** Yi-Yu Hu, Yao-Ping Xie, Lu Wu, Jian-Tao Qin, Rong-Jian Pan, Mei-Yi Yao

**Affiliations:** 1 School of Materials Science and Engineering, Shanghai University, Shanghai 200444, China; huyiyu@shu.edu.cn (Y.-Y.H.); yaomeiyi@shu.edu.cn (M.-Y.Y.); 2 The First Sub-Institute, Science and Technology on Reactor Fuel and Materials Laboratory, Nuclear Power Institute of China, Chengdu 610005, China; wulu1002@126.com (L.W.); qjtuestc@163.com (J.-T.Q.); haoyunjiuzhe2008@126.com (R.-J.P.)

**Keywords:** first-principles calculations, ferritic/martensitic steels, swelling, irradiation defects, diffusion

## Abstract

Ferritic/martensitic (F/M) steels whose matrix is Fe-Cr are important candidate materials for fuel cladding of fast reactors, and they have excellent irradiation-swelling resistance. However, the mechanism of irradiation-swelling of F/M steels is still unclear. We use a first-principles method to reveal the influence of irradiation defects, i.e., Frenkel pair including atomic vacancy and self-interstitial atom, on the change of lattice volume of Fe-13Cr lattice. It is found that vacancy causes lattice contraction, while a self-interstitial atom causes lattice expansion. The overall effect of a Frenkel pair on the change of lattice volume is lattice expansion, leading to swelling of the alloy. Furthermore, the diffusion properties of point defects in Fe-13Cr are investigated. Based on the diffusion barriers of the vacancies and interstitial atoms, we find that the defects in Fe-13Cr drain out to surfaces/grain boundaries more efficiently than those in pure α-Fe do. Therefore, the faster diffusion of defects in Fe-13Cr is one of important factors for good swelling resistance of Fe-13Cr compared to pure α-Fe.

## 1. Introduction

Ferritic/martensitic (F/M) steels have excellent high-temperature strength, creep-rupture strength, corrosion resistance, neutron irradiation resistance, and low price, which are promising candidates for the new generation of cladding materials of fast reactors [1,2]. One of the important characteristics of F/M steels is its excellent swelling resistance during neutron irradiation, which is much better than that of austenitic steels, the first-generation cladding materials for fast reactors. For example, when neutron fluence is more than 16 × 10^22^ n/cm^2^, the swelling rate of F/M steels is 1/5 to 1/25 of that of austenitic steels [3]. However, the mechanism of radiation-swelling resistance of F/M steels is not clear and therefore attracts much attention.

To reveal the swelling mechanism of F/M steels, many works have been dedicated to extensively investigating the correlation between the microstructure and swelling behavior of Fe-Cr alloys, and the dependence of temperature, irradiation dose, and alloy composition on the swelling behavior [4]. Gelles performed microstructural examination on binary Fe-Cr alloys irradiated in Fast Flux Test Facility Materials Open Test Assembly (FFTF/MOTA) [5]. Dislocation networks and well-developed voids were found in the high swelling sample, and the swelling rate depends on the alloy composition. Bhattacharya et al., performed self-ions irradiation on Fe-Cr alloys, and they found that the alloy swelling can be suppressed by addition of Cr, which is achieved by a dramatic reduction of cavity size [6]. 

Zheng et al., used in situ TEM to study the commercial F/M steels HT9, and they found that the size and density of dislocation loops and dislocation lines depends on dose and temperature [7]. Furthermore, they performed irradiation on HT9 in the BOR-60 reactor, and microstructural examination showed that the size of the cavities depends on the irradiation temperature. They also confirmed that the large degree of swelling is due to the presence of large cavities [8].

To deeply understand microstructure evolution of F/M steels under irradiation, extensive atomic simulations have been performed [9]. Under irradiation conditions, some atoms are knocked off from the lattices, which are usually named primary knock-on atoms (PKA), to form Frenkel pairs, and then form damage cascade zones. To investigate the relevance between these processes and irradiation performances, extensive studies have been performed by using molecular dynamics (MD). Terentyev et al., used MD to investigate displacement cascades in Fe-10 at.%Cr alloys, and they showed that the proportion of Cr atoms at the interstitial positions is relatively large [10]. Abu-Shams et al., studied primary irradiation damage of Fe-10 at.%Cr under uniaxial, biaxial, and hydrostatic pressures. They found that Cr atoms prefer to occupy interstitial positions in all cases [11,12].

Since first-principles calculation is more accurate and not limited by interatomic potential, there are also first-principles investigations on irradiation effects of Fe-Cr alloys. Nguyen-Manh et al., studied the interaction of vacancies with alloying elements such as V, Nb, Ta, Mo, W, Al, Si, P, and S [13]. Martinez et al., studied the clustering of vacancies with He and their interaction with Cr, revealing the role of He in micro-voids [14]. Senninger et al., analyzed the mechanism of the segregation behavior of Cr [15]. Ding et al., studied the stability of vacancy clusters and the effect of He on vacancy stability in Fe-9 wt.%Cr and Fe-based alloys, which provided a thermodynamic basis for understanding vacancy accumulation and vacancy cluster growth [16].

As mentioned above, the microstructure evolution under irradiation conditions has been widely investigated. The effect of irradiation defects on the volume of the F/M steels is an important factor in understanding swelling effect, but the reports on this topic are still few, which limit the understanding of the mechanism of F/M swelling under irradiation conditions. In order to optimize the irradiation-swelling resistance of F/M alloys rationally, it is important to understand the effect of typical defects on the volume of the F/M alloys under irradiation conditions. Since first-principles calculation can provide reliable structural and thermodynamic properties of defects in materials, we perform first-principles calculation to investigate the thermodynamic and kinetic properties of Frenkel pairs in F/M steels, including the stability of the defects, the influence of defects on the lattice volume and the diffusion barriers of defects, and analyze their effects on irradiation swelling.

## 2. Atomic Models and Methods

**Figure 1:**
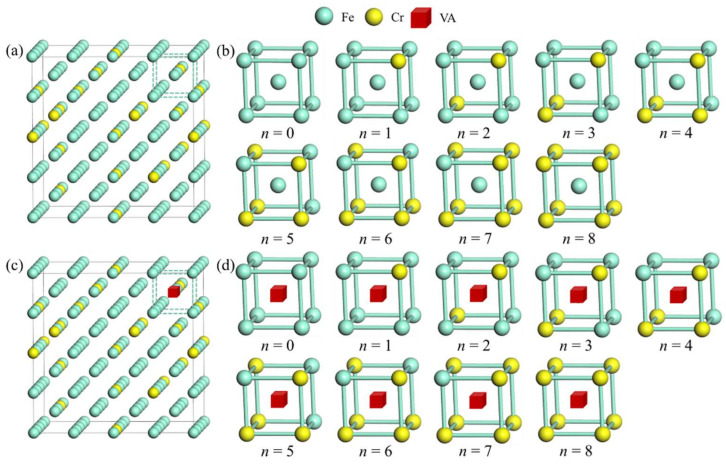
Illustration of atomic structures for the Fe-13Cr supercells with and without a vacancy. (**a**) A 128-atom supercell containing 111 Fe atoms and 17 Cr atoms; (**b**) atomic structures with different numbers of Cr atoms in dashed line box; (**c**) a 127-atom supercell with a vacancy containing 110 Fe atoms and 17 Cr atoms; (**d**) atomic structures in dashed line boxes for vacancies with different numbers of nearest-neighbor Cr atoms.

A first-principles method based on density functional theory (DFT) is used for the total energy calculations, which are carried out using the Vienna ab initio simulation package (VASP package, version 5.3) [17,18]. The interaction between ions and valence electrons is described by Projector-Augmented Wave (PAW) [19,20]. A generalized gradient approximation (GGA) [21,22,23] under the Perdew–Burke–Ernzerhof (PBE-PAW version 54) functional is used to handle the electron exchange correlations [24,25,26]. As the method that Ding et al. [16] used to model the Fe-9Cr alloy, a stochastic solid solution method is used to generate a Fe-13Cr binary alloy model containing 128 atoms (111 Fe atoms and 17 Cr atoms), which is shown in Figure 1a.

The energy cut-off of plane-wave basis is set as 450 eV for all calculations. The k-points of the Brillouin zone are sampled using the Monkhorst–Pack method [27]. The k-point grid is set to 2 × 2 × 2 for Fe-13Cr model supercell. The convergence criterion for self-consistent calculations is a change in total energy between two steps less than 1 × 10^−5^ eV for electron relaxation and 0.01 eV for ion relaxation. Spin polarization calculations are used in all of the calculations. The size and shape of the supercell, as well as the atoms in the supercell, are all optimized in structure relaxation. The calculated lattice constants for the Fe, Cr, and Fe-13Cr of BCC structure are listed in Table 1. The calculated results are consistent with the previous experimental values and DFT results [28,29,30,31].

Figure 1 shows the atomic structures for supercells describing Fe-13Cr with and without atomic vacancy. In Fe-13Cr, the vacancies can be classified by its nearest-neighbor number of Cr atoms. To investigate the stability of vacancies with different numbers of nearest-neighbor Cr atoms, we construct two types of supercells.

First, we select a Fe site in a supercell, which is marked by green dashed lines in Figure 1a, and exchange its nearest-neighbor atoms with Fe or Cr in other sites to obtain different supercells to model different vacancies. The corresponding zone of dashed line cubic in different supercells are plotted in Figure 1b, which clearly shows different nearest-neighbor atoms around the Fe site.

Second, using the above-mentioned supercells, we delete the Fe atom in the central site of the green dashed-line cube and obtain supercells containing vacancies with different numbers of nearest-neighbor Cr atoms. The corresponding zones of dashed line cubes in different supercells are plotted in Figure 1d, which clearly shows atomic vacancy with different numbers (*n*) of nearest-neighbor Cr atoms. For convenience, we denote the atomic vacancy with the number of nearest-neighbor Cr atoms *n* as $V^{n\mathrm{Cr}}$.

The vacancy formation energy $E_{V^{n\mathrm{Cr}}}^{f}$ can be calculated from the following equation:

(1) $$E_{V^{n\mathrm{Cr}}}^{f}=\left(E_{V^{n\mathrm{Cr}}}+\mu_{\mathrm{Fe}}\right)-E_{n\mathrm{Cr}}$$

where $E_{V^{n\mathrm{Cr}}}$ represents the total energy of supercell with a vacancy $V^{n\mathrm{Cr}}$ and $E_{n\mathrm{Cr}}$ is the total energy of the corresponding supercell containing Fe-13Cr lattice without vacancy. The supercell with vacancy just has one less Fe atom than that of the corresponding Fe-13Cr lattice supercell. $\mu_{\mathrm{Fe}}$ is the chemical potential of one Fe atom, which is the total energy per atom in the pure α-Fe [32].

The influence of the vacancy on the volume of the alloy can be reflected by $V_{V^{n\mathrm{Cr}}}^{d}$, which is the volume difference between supercell with and without a vacancy. The value of $V_{V^{n\mathrm{Cr}}}^{d}$ is computed by the following equation:

(2) $$V_{V^{n\mathrm{Cr}}}^{d}=V_{V^{n\mathrm{Cr}}}-V_{n\mathrm{Cr}}$$

where $V_{V^{n\mathrm{Cr}}}$ denotes the volume of the supercell containing one vacancy and $V_{n\mathrm{Cr}}$ denotes the volume of the corresponding Fe-13Cr supercell without vacancy.

**Figure 2:**
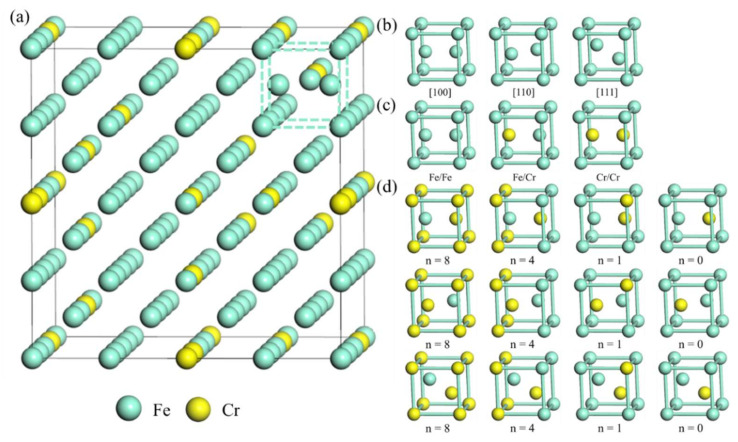
Illustration of atomic structure for the Fe-13Cr supercell containing a dumbbell structure of self-interstitial atoms: (**a**) a 129-atoms supercell with a dumbbell structure containing 112 Fe atoms and 17 Cr atoms; (**b**) atomic structures of Fe/Fe dumbbell structure along different crystal orientations in dashed line box; (**c**) atomic structures of [110] dumbbell structure with different compositions in dashed line box; (**d**) atomic structures of dumbbell structure with different numbers of nearest-neighbor Cr atoms in dashed line box.

There many possible atomic configurations of dumbbells that comprise self-interstitial atoms. First, the dumbbells have many crystal orientations; here, we only consider 3 typical orientations, i.e., [100], [110], and [111], and we plot the atomic structures of the self-interstitial atoms consisting of Fe atoms along these orientations in Figure 1a. Second, the dumbbell also has 3 possible compositions. Figure 2b gives the [100] dumbbell structures with different compositions. Third, the dumbbell has 9 possible numbers of nearest-neighbor Cr atoms; here, we only consider 4 typical types, i.e., dumbbell structures with the numbers nearest-neighbor Cr atoms of 8, 4, 1, and 0. Figure 2c shows Fe/Cr dumbbell structures along [100], [110], and [111] with the numbers of nearest-neighbor Cr atoms 8, 4, 1, and 0 respectively. For convenience, we denote the dumbbell structure as M1/M2[mnl]*^n^*^Cr^, where M1 and M2 is the type of interstitial atoms in dumbbell, and [mnl] is the crystal orientation of dumbbell. For example, the dumbbell containing a Fe and a Cr atom along [110] with 1 nearest-neighbor Cr atom is denoted as Fe/Cr [110]^1Cr^.

The self-interstitial atom formation energy ($E_{M1/M2{\left[\mathrm{mnl}\right]}^{n\mathrm{Cr}}}^{f}$) can be calculated from the following equation:

(3) $$E_{M1/M2{\left[\mathrm{mnl}\right]}^{n\mathrm{Cr}}}^{f}=E_{M1/M2{\left[\mathrm{mnl}\right]}^{n\mathrm{Cr}}}-\left(E_{n\mathrm{Cr}}+n_{\mathrm{Fe}}\mu_{\mathrm{Fe}}+n_{\mathrm{Cr}}\mu_{\mathrm{Cr}}\right)$$

where $E_{M1/M2{\left[\mathrm{mnl}\right]}^{n\mathrm{Cr}}}$ represents the total energy of supercell with $M1/M2{\left[\mathrm{mnl}\right]}^{n\mathrm{Cr}}$, $E_{n\mathrm{Cr}}$ represents the total energy of the corresponding Fe-13Cr supercell, and $n_{\mathrm{Fe}}$ and $n_{\mathrm{Cr}}$ are the number difference of Fe and Cr atoms between supercells with and without $M1/M2{\left[\mathrm{mnl}\right]}^{n\mathrm{Cr}}$ structure, respectively.

The effect of the formation of self-interstitial atoms on the volume of the alloy is described by the volume difference $V_{M1/M2{\left[\mathrm{mnl}\right]}^{n\mathrm{Cr}}}^{d}$ between the supercell containing the self-interstitial atom and its counterpart, the Fe-13Cr supercell, which is defined as follows:

(4) $$V_{M1/M2{\left[\mathrm{mnl}\right]}^{n\mathrm{Cr}}}^{d}=V_{M1/M2{\left[\mathrm{mnl}\right]}^{n\mathrm{Cr}}}-V_{n\mathrm{Cr}}$$

where $V_{M1/M2{\left[\mathrm{mnl}\right]}^{n\mathrm{Cr}}}$ denotes the volume of supercell with $M1/M2{\left[\mathrm{mnl}\right]}^{n\mathrm{Cr}}$, and $V_{n\mathrm{Cr}}$ denotes the volume of the corresponding Fe-13Cr supercell.

To investigate the trend of clustering of atomic vacancies, we also compute the formation energy of the vacancy cluster, which is composed of several atomic vacancies. We denote vacancy cluster with *m* atomic vacancies as $V_{m}$. We also construct a corresponding supercell whose structure is the same as ${V_{m}}_{\;}$except in vacancy cluster zone, and it is named $V_{m}^{\prime }$.

The formation energy of $V_{m}$ is defined as follows:

(5) $$E_{V_{m}}^{f}=(E_{V_{m}}-E_{V_{m}^{\prime }}+n_{\mathrm{Fe}}\mu_{\mathrm{Fe}}+n_{\mathrm{Cr}}\mu_{\mathrm{Cr}})/m$$

where $E_{V_{m}}$ represents the total energy of supercell with $V_{m}$, $E_{V_{m}^{\prime }}$ represents the total energy of the corresponding Fe-13Cr supercell, $\mu_{\mathrm{Cr}}$ is the chemical potential of one Cr atom, which is the total energy per atom in the pure α-Cr supercell, and $n_{\mathrm{Fe}}$ and $n_{\mathrm{Cr}}$ are the number difference of Fe and Cr atoms between supercells with and without $V_{m}$, respectively. 

The volume of the alloy induced by vacancy clusters is described by $V_{V_{m}}^{d}$ and it is defined as follows:

(6) $$V_{V_{m}}^{d}=(V_{V_{m}}-V_{V_{m}^{\prime }})/m$$

where $V_{V_{m}}$ and $V_{V_{m}^{\prime }}\;$represent the volume of supercell with and without $V_{m}$, respectively. 

To reveal the diffusion properties of vacancy and interstitial atoms, we also calculate their diffusion energy barrier. Here, we use a newly developed and high-efficiency stochastic surface walking (SSW) method [33]. It can sample the potential energy surface (PES) of the atomic structure and find the lowest pathway of atomic structural evolution. In the frame of SSW, we use the double-ended surface walking (DESW) [34] method to compute the diffusion path of vacancy and the self-interstitial atom. Using this method, starting from the initial and final states of the atomic structures, the atoms of the two structures move towards each other by gradually adding the form of the Gaussian function until they obtain the same structure, and then the pseudo-pathway is established. The structure with the highest energy of pseudo-pathway is selected, the biased rotation of the constrained Broyden dimer rotation (CBD) method is adopted, and the transition state (TS) is accurately determined on the reaction path. After obtaining TS, the diffusion barrier is calculated by the following equations:

(7) $$E_{EB}=E_{TS}-E_{IS}$$

where $E_{TS}$ is the total energy of the transition state and $E_{IS}$ is the total energy of the initial state.

## 3. Results and Discussions

### 3.1. The Atomic Vacancies

**Figure 3:**
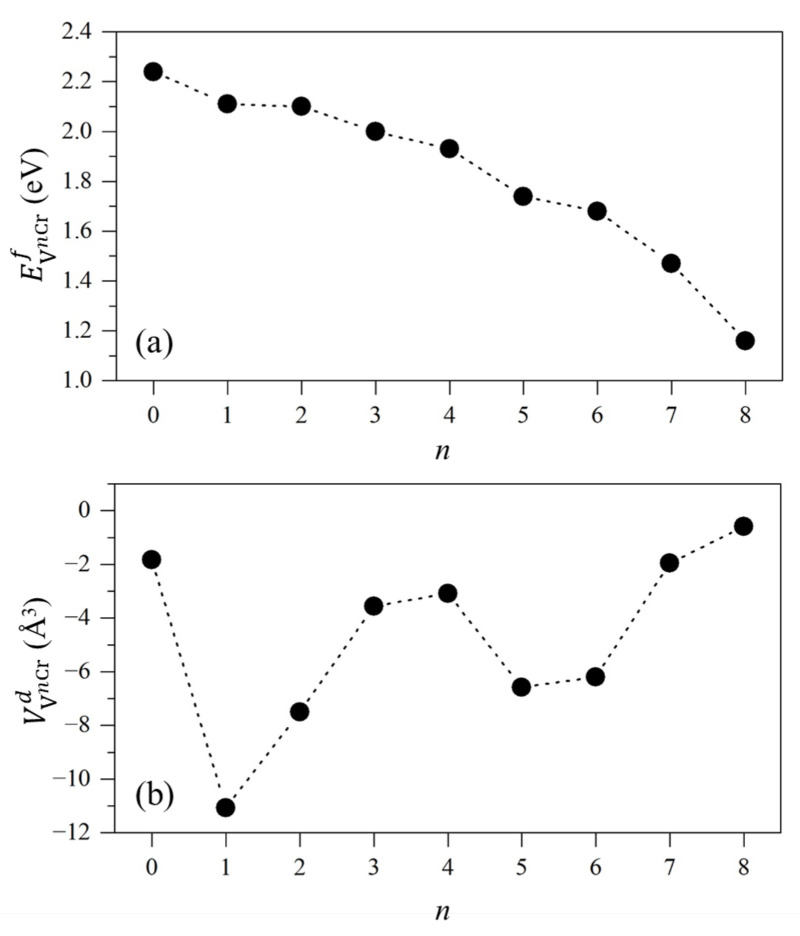
(**a**) Formation energy ($E_{V^{n\mathrm{Cr}}}^{f}$) of atomic vacancy, and (**b**) volume difference ($V_{V^{n\mathrm{Cr}}}^{d}$) between supercell with and without a vacancy for a given *n*. *n* is the numbers of nearest-neighbor Cr atoms around vacancy.

The formation energies of atomic vacancies in Fe-Cr alloys are shown in Figure 3a, and their values are in the range of 1.16~2.24 eV. The formation energies of vacancies decrease with increasing number of nearest-neighbor Cr atoms. As is well known, there are two phases, i.e., α and $\alpha^{\prime }$, in the F/M alloys with 13 at.% Cr, and the Cr contents of α phases are among 10~13 at.%, while those of $\alpha^{\prime }$ phases are among 60~80 at.% under 250~520 °C [35,36,37]. If the Fe and Cr atoms are randomly distributed in alloys, the most prevailing vacancies in the α-phase are the vacancies with one nearest-neighbor Cr atom. Therefore, as shown in Figure 3a, the vacancy formation energy in the α-phase is 2.11 eV, which is the same as that in α-Fe, namely 2.11 eV. It indicates that the formation difficulty of atomic vacancy in the α phase of Fe-13Cr is almost the same as that in α-Fe. The most prevailing vacancies in the $\alpha^{\prime }$-phase are the vacancies with six nearest-neighbor Cr atoms. Therefore, as shown in Figure 3a, the formation energy of vacancies in $\alpha^{\prime }$ phase is 1.68 eV, which is lower than that in α-Fe. It indicates that the formation of atomic vacancies in $\alpha^{\prime }$-phase is easier than that in α-Fe. Since α phase is the main constituent in Fe-13Cr, the difficulty of vacancy formation in Fe-13Cr is similar to that in α-Fe.

Figure 3b shows the influences of atomic vacancies on the lattice volume of Fe-13Cr. It shows that vacancies induce a volume reduction effect on Fe-13Cr lattice. The values of volume difference induced by an atomic vacancy of Fe-13Cr are between −0.60 and −11.07 Å^3^. The relationship between the reduction in volume and the number of nearest-neighbor Cr atoms for an atomic vacancy is non-monotonic. Nevertheless, from all of results, the vacancy generally induces a volume reduction effect of Fe-13Cr. The sensitivity of volume difference of Fe-13Cr on nearest-neighbor Cr atoms of atomic vacancy is caused by the complicated magnetic coupling between atoms around the vacancy. Similar complicated changes of alloy lattice induced by magnetic properties also were found in INVAR alloy which is Fe-Mn based alloy [38].

The prevailing atomic vacancy is $V^{1\mathrm{Cr}}$, whose nearest-neighbor Cr atoms is 1. The volume reduction induced by an $V^{1\mathrm{Cr}}$ in Fe-13Cr is 11.07 Å^3^. To check the influence of supercell size on the results of formation energy and volume difference, we have computed $V^{1\mathrm{Cr}}$ using a supercell with 64, 128 and 250 atoms. Moreover, we check the $V^{1\mathrm{Cr}}$ with different local atomic configuration; i.e., we fix the nearest-neighbor Cr atom as 1 and change the Cr occupied site in the supercell to obtain different structures. We present the formation energies and volume difference of these structures of $V^{1\mathrm{Cr}}$ in Table 2.

It is found that the values of formation energy of $V^{1\mathrm{Cr}}$ are not sensitive to the supercell size and local atomic structures, while the values of volume difference of $V^{1\mathrm{Cr}}$ is very sensitive to supercell size and local atomic structures. The values of volume difference of $V^{1\mathrm{Cr}}$ are dispersed, and they do not converge with the increase in supercell size. Therefore, we can infer that the dispersion of these values derives from the sensitivity of lattice volume to magnetism that is influenced by the local atomic structure of vacancies. 

From Table 1, we can obtain that the values of volume reduction of $V^{1\mathrm{Cr}}$ are between −2.57 and −11.07 Å^3^. Therefore, though values of volume difference of $V^{1\mathrm{Cr}}$ are dispersed, all of the results are negative, indicating that it has an effect of lattice volume reduction. The value of volume difference induced by an atomic vacancy in α-Fe is −3.22 Å^3^. Therefore, vacancies play the same role in both Fe-13Cr and α-Fe, inducing lattice reduction.

### 3.2. Self-Interstitial Atoms

**Figure 4:**
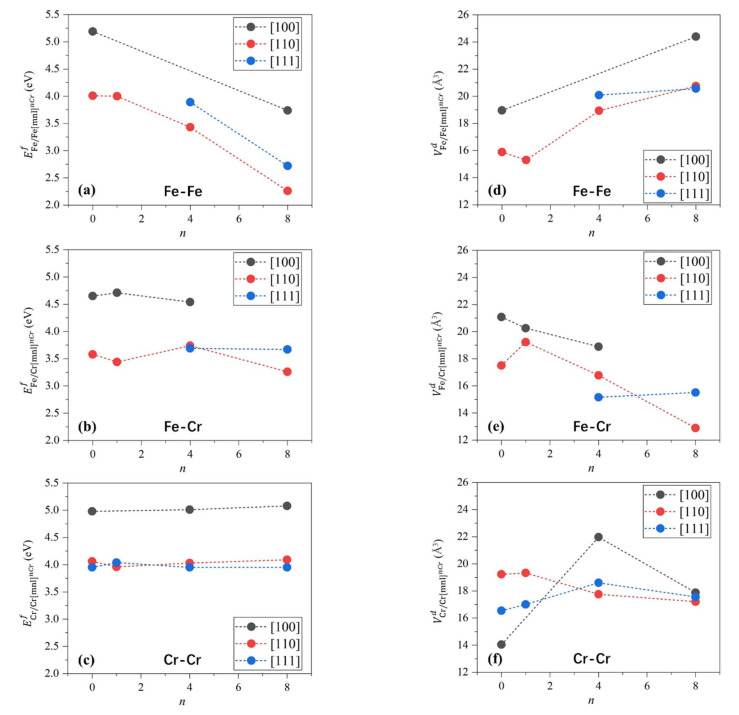
(**a**,**b**,**c**) The formation energies of dumbbells different orientations, compositions, and nearest-neighbor Cr atoms. Some of dumbbells do not even have meta-stability, and therefore the formation energies of these structures are absent. (**d**,**e**,**f**) The influence of different dumbbells on the lattice volume. The dashed lines are just to guide the eyes.

We compute the formation energies of dumbbells composed of self-interstitial atoms with different orientations, compositions, and nearest-neighbor Cr atoms, and the results are listed in Figure 4. The formation energies of $\mathrm{Fe}/\mathrm{Fe}{\left[\mathrm{mnl}\right]}^{n\mathrm{Cr}}$, $\mathrm{Fe}/\mathrm{Cr}{\left[\mathrm{mnl}\right]}^{n\mathrm{Cr}}$, and $\mathrm{Cr}/\mathrm{Cr}{\left[\mathrm{mnl}\right]}^{n\mathrm{Cr}}$ are between 2.26 and 5.19, 3.26 and 4.65, and 3.74~4.98 eV, respectively. The formation energies of the $\mathrm{Fe}/\mathrm{Fe}{\left[\mathrm{mnl}\right]}^{n\mathrm{Cr}}$ and $\mathrm{Fe}/\mathrm{Cr}{\left[\mathrm{mnl}\right]}^{n\mathrm{Cr}}$ are relatively lower. The formation energy of $\mathrm{Fe}/\mathrm{Fe}{\left[110\right]}^{8\mathrm{Cr}}$ is 2.26 eV/dumbbell, which is the lowest.

However, since the concentration of Cr in Fe-13Cr is 13 at.%, the number of atomic configurations of dumbbells with eight nearest-neighbor Cr atoms is limited, while the dumbbells with one nearest-neighbor Cr atom are dominant. For dumbbells with one nearest-neighbor Cr atom, the most thermodynamically favorable configuration is $\mathrm{Fe}/\mathrm{Cr}{\left[110\right]}^{1\mathrm{Cr}}$, whose formation energy is 3.44 eV/dumbbell. Therefore, considering both formation energy and alloy composition, we infer that the most common configuration of dumbbell structure is $\mathrm{Fe}/\mathrm{Cr}{\left[110\right]}^{1\mathrm{Cr}}.$ These findings are consistent with molecular dynamics results by Vörtler et al., in which the Fe/Cr[110] dumbbells were dominant in the Fe-10 at.%Cr matrix after irradiation [39]. 

Figure 4 shows the influence of different dumbbells on the lattice volume. All of the dumbbells cause lattice expansion. The increase of the lattice volume by a single dumbbell is between 12 and 25 Å^3^. The largest lattice expansion is induced by $\mathrm{Fe}/\mathrm{Fe}{\left[110\right]}^{8\mathrm{Cr}}$, and its volume increase is 24.29 Å^3^/dumbbell. The smallest lattice expansion is induced by $\mathrm{Cr}/\mathrm{Cr}{\left[100\right]}^{8\mathrm{Cr}}$, and its volume increase is 12.73 Å^3^/dumbbell. For the most common dumbbell, $\mathrm{Fe}/\mathrm{Cr}{\left[110\right]}^{1\mathrm{Cr}}$, the volume increase is 19.23 Å^3^/dumbbell. Furthermore, we have checked the influence of the supercell size on the formation energy of $\mathrm{Fe}/\mathrm{Cr}{\left[110\right]}^{1\mathrm{Cr}}$ and its influence on volume change. As shown in Table 3, the formation energies from the calculation by different supercell sizes are close to each other, as are the values of the volume difference.

$E_{SIA}^{f}$$V_{SIA}^{d}$ Vacancies lead to lattice reduction and self-interstitial atoms lead to lattice expansion. For Fe-13Cr, the most common dumbbell, $\mathrm{Fe}/\mathrm{Cr}{\left[110\right]}^{1\mathrm{Cr}}$, leads to a lattice increase of 19.23 Å^3^/dumbbell, while the most common vacancy, $V^{1\mathrm{Cr}}$, leads to a lattice reduction of 2.57~11.07 Å^3^/Vacancy. Therefore, an irradiation-induced Frenkel pair results in an expansion of 8.16~16.66 Å^3^/pair. For α-Fe, the [110] dumbbell leads to a lattice increase of 18.91 Å^3^/dumbbell, and the vacancy leads to a lattice reduction of 3.22 Å^3^/V. Therefore, the irradiation-induced Frenkel defect results in an expansion of 15.69 Å^3^/pair. From these data, we can infer that Frenkel will lead to the lattice expansion of both Fe-Cr and α-Fe alloys.

### 3.3. Vacancy Cluster

**Figure 5:**
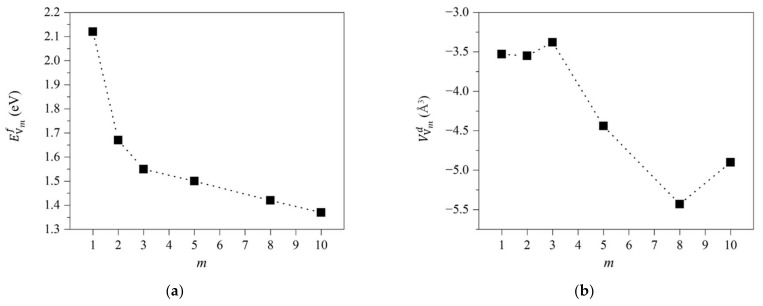
(**a**) The formation energies of vacancy clusters with different sizes and (**b**) the influence on lattice volume.

Since the voids (also named cavities) were frequently observed in the irradiated and swelling F/M steels, we attempt to understand the gathering of atomic vacancy, which is the very initial stage of void formation. Limited by the computational expenditure of first-principles calculations, we only consider the vacancy clusters composed of 2~10 vacancies. We delete the several neighbor atoms in the 128-atom supercell to construct vacancy clusters and compute the formation energy using Equation (5). Figure 5a shows the dependence of formation energy on the size of vacancy cluster. The formation energies of vacancy-clusters decrease with their sizes, indicating that atomic vacancies tend to form atomic cluster. As shown in Figure 5b, the vacancy clusters also induce lattice volume reduction. The dependence of volume change on cluster size is also non-monotonic.

**Figure 6:**
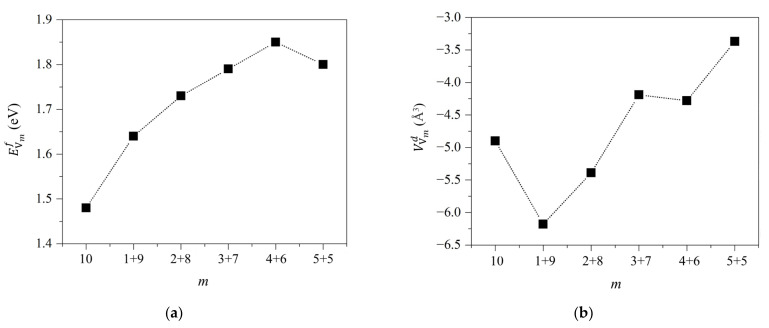
(**a**) The formation energies of two vacancy clusters and (**b**) the influence of vacancy-clusters on lattice volume change.

To compare the relative stability of larger and smaller clusters, we construct a cluster with 10 vacancies, and a series of combination of two clusters with N1 and N2(N1+N2=10) vacancies respectively, i.e., N1 = 1, N2 = 9; N1 = 2, N2 = 8; N1 = 3, N2 = 7; N1 = 4, N2 = 6; N1 = 5, and N2 = 5. Figure 6a shows the formation energies of these clusters. It indicates that the cluster with 10 vacancies is the most stable. The stability of combinations of two clusters roughly increases with the size of the larger one. These findings also indicate that the cluster is likely to grow larger. As shown in Figure 6b, the vacancy clusters also induce lattice volume reduction. The dependence of volume change on cluster size is still non-monotonic.

### 3.4. Diffusion Properties of Defects

**Figure 7:**
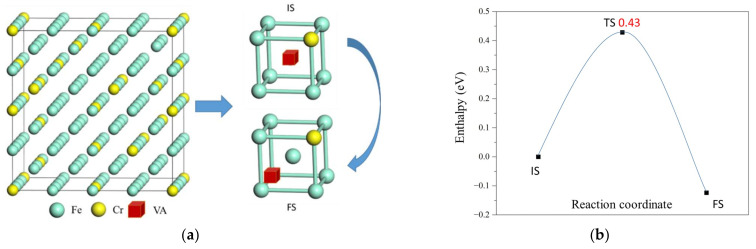
(**a**) Illustration of atomic structure for vacancy diffusion pathway and (**b**) energy profile for vacancy diffusion in $V^{1\mathrm{Cr}}$. IS is the initial state, TS is the transition state, and FS is the final state of the diffusion.

**Figure 8:**
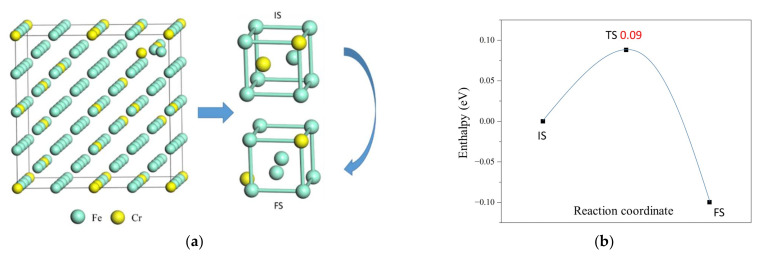
(**a**) Illustration of atomic structure for self-interstitial atoms diffusion pathway and (**b**) energy profile for self-interstitial atom diffusion in $\mathrm{Fe}/\mathrm{Cr}{\left[110\right]}^{1\mathrm{Cr}}$.

The kinetic properties of defects are also important factors to influence the microstructure of F/M steels under irradiation. We compute the diffusion barriers of vacancy and self-interstitial atoms in Fe-13Cr using the DESW and DFT methods. Figure 7a and Figure 8a show a step of $V^{1\mathrm{Cr}}$ vacancy and $\mathrm{Fe}/\mathrm{Cr}{\left[110\right]}^{1\mathrm{Cr}}$ self-interstitial atom diffusion in the lattice, respectively, and Figure 7b and Figure 8b show the energy profiles on the diffusion pathway of vacancy and self-interstitial atom, respectively. It is shown only one transition state between two structures with vacancy (self-interstitial atom) at two different lattice sites. The vacancy (self-interstitial atom) at different lattices has different energy; i.e., the IS and FS in Figure 7a (Figure 8a) have different energies. This is because different lattice sites have different numbers of nearest-neighbor Cr atoms.

Table 4 shows the comparison of diffusion barriers and diffusion pathways of vacancies and self-interstitial atoms in Fe-13Cr and α-Fe. Here, we mainly compute the diffusion pathway of vacancies (or self-interstitial atoms) between the site $V^{1\mathrm{Cr}}$(or $\mathrm{Fe}/\mathrm{Cr}{\left[110\right]}^{1\mathrm{Cr}}$) because the commonly existing vacancy and the self-interstitial atom in Fe-13Cr both have a *n* value of 1. Therefore, the diffusion barriers of the pathway between these sites should have large effects on the rate of defect diffusion. We also examine some diffusion barriers between sites with other values of *n.*

The diffusion barriers of vacancies in Fe-13Cr are between 0.35 and 0.66 eV, which are all smaller than that of vacancies in pure α-Fe, 0.74 eV. This indicates that the diffusion rate of vacancies in Fe-13Cr is larger than that of vacancies in pure α-Fe. The diffusion barriers of self-interstitial atoms in Fe-13Cr are between 0.09 and 0.49 eV, while those of self-interstitial atoms in pure α-Fe is 0.32 eV. The diffusion barriers of self-interstitial atoms with *n*=1 in Fe-13Cr are between 0.21 and 0.31 eV, and the values of most pathways are lower than those of self-interstitial atoms in pure α-Fe, which can provide easy pathway for diffusion of self-interstitial atoms. These finding indicate that the diffusion rate of self-interstitial atoms in Fe-13Cr is also larger than that of vacancies in pure α-Fe. Since the defects in Fe-13Cr are faster, and therefore there is an annihilation process of defects such as recombination of voids and self-interstitial atoms, the defects draining out to the surface/grain boundaries in Fe-13Cr are faster compared to pure α-Fe.

After obtaining a series of thermodynamic and kinetic properties of irradiation defects in Fe-13Cr, we can discuss the irradiation swelling mechanism of this alloy. The influence of irradiation defects on irradiation swelling can be analyzed in terms of three aspects. First, the formation energy of commonly existing atomic vacancy in Fe-13Cr alloy is the same as that in pure α-Fe, while the formation energy of the commonly existing interstitial atom in Fe-13Cr is slightly lower than that of interstitial atom in pure α-Fe. Therefore, the formation of a Frenkel pair in Fe-13Cr alloy is easier than in pure α-Fe, which is not a conducive factor contributing to the swelling resistance of Fe-13Cr being better than that of pure α-Fe. Second, the volume change of Fe-13Cr induced by the Frenkel pair has a wide range, while the volume change of pure α-Fe induced by the Frenkel pair is close to the maximum of these for Fe-13Cr. This indicates that volume expansion induced by the Frenkel pair has a chance of being smaller than that of pure α-Fe, which is a conducive factor contributing to the swelling resistance of Fe-13Cr being better than that of pure α-Fe. Third, the diffusion barriers of the vacancy and the interstitial atom in Fe-13Cr alloy are much smaller than those of the vacancy and the interstitial atom in pure α-Fe, respectively, so the diffusion rate of vacancy and interstitial atom in Fe-13Cr is much larger than that in pure α-Fe, which leads to the vacancy and interstitial atom crashing into the grain boundary and the surface of the alloys to more efficiently reduce the defect concentration. From all the above, one of the important factors for better swelling resistance of Fe-13Cr compared to pure α-Fe is that the diffusion rate of defects in Fe-13Cr alloy is larger than that in α-Fe.

It is frequently reported that a large swelling of Fe-Cr alloy corresponds to void formation in the matrix, and the irradiation swelling of Fe-Cr alloys is also referred to as void swelling [4]. From our results, we can infer that the annihilation process of defects in Fe-13Cr is faster than that in pure α-Fe, which leads to the possibility of void formation and growth in Fe-13Cr being smaller than in pure α-Fe. Therefore, the void-swelling resistance of Fe-13Cr is better than that of pure α-Fe. Though we can partly identify the factors that are benefit to swelling resistance of Fe-13Cr, it is still necessary to combine the statistical data of thermodynamics and kinetics, which can be completed by molecular dynamics using high-precision interatomic potential in the future.

## 4. Conclusions

The thermodynamic and kinetic properties of irradiation defects that are related to swelling of Fe-Cr alloys are investigated. The following conclusions can be drawn:

1. The formation energy of the commonly existing vacancy in Fe-13Cr is 2.11 eV, which is the same with that in pure α-Fe, indicating that the difficulty of vacancy formation in α-Fe and Fe-13Cr is close. The formation energy of the commonly existing self-interstitial atoms of dumbbell, $\mathrm{Fe}/\mathrm{Cr}{\left[110\right]}^{1\mathrm{Cr}}$, in Fe-13Cr is 3.44 eV, which is lower than that of self-interstitial atoms in pure α-Fe, 3.93 eV, and this is a harmful factor to the swelling resistance of Fe-13Cr being better than that of pure α-Fe.
2. The formation energy of the vacancy cluster increases with its size, indicating that the vacancy cluster tends to grow larger from the perspective of thermodynamics.
3. The vacancy and vacancy cluster induce volume reduction, while a self-interstitial atom induces volume expansion for Fe-13Cr alloys. The influence of a Frenkel pair induces the expansion of lattice volume for Fe-13Cr. The expansion of Fe-13Cr induced by Frenkel pairs is between 8.16 and 16.66 Å^3^/pair, while the expansion of pure α-Fe induced by Frenkel pairs is 15.69 Å^3^/pair. There is the possibility for Frenkel pair to induce smaller lattice expansion of Fe-13Cr compared to pure α-Fe, which is a conducive factor contributing to the swelling resistance of Fe-13Cr being better than that of pure α-Fe.
4. Based on the data of diffusion barriers, we found that the diffusion rates of vacancy and self-interstitial atoms in Fe-13Cr are larger than those of the vacancy and the self-interstitial atom in pure α-Fe. Therefore, the annihilation process of these defects in Fe-13Cr is faster than in pure α-Fe, which increases the swelling resistance compared to pure α-Fe.

## Figures and Tables

**Table 1:** Comparison of calculated and experimental values of lattice constants for pure α-Fe, pure α-Cr, and Fe-13Cr alloys (Å).

	This Work	DFT
Pure α-Fe	2.83	2.83 [30], 2.84 [28]
Pure α-Cr	2.87	2.85 [30], 2.85 [28]
Fe-13Cr	2.84, 2.83 *	2.84 [31], 2.85 [28]

* the calculated lattice lengths of Fe-Cr supercell are not exactly the same due to random distribution of Cr at lattice.

**Table 2:** Comparison of the formation energies of the atomic vacancy $V^{1\mathrm{Cr}}$ in supercells with 54, 128, and 250 atoms, and the volume difference of supercell caused by an atomic vacancy. We mark the structures of $V^{1\mathrm{Cr}}$ with different local atomic configurations by labels 1, 2, and 3.

Number of Atoms		54	128	250
$E_{V}^{f}$ (eV)	labels 1 labels 2 labels 3	2.08 2.16 2.17	2.11 2.21 2.24	2.06 2.12 2.17
$V_{V}^{d}$ (Å^3^)	labels 1 labels 2 labels 3	−6.07 −3.84 −4.88	−11.07 −3.00 −2.76	−2.57 −7.19 −4.40

**Table 3:** Comparison of the formation energies of $\mathrm{Fe}/\mathrm{Cr}{\left[110\right]}^{1\mathrm{Cr}}$ with 54, 128, and 250 atoms, and the volume difference of supercell caused by a self-interstitial atom.

Number of Atoms	54	128	250
$E_{SIA}^{f}$ (eV)	3.65	3.44	3.65
$V_{SIA}^{d}$ (Å^3^)	18.39	19.23	19.00

**Table 4:** The diffusion barriers of $V^{n\mathrm{Cr}}$ and $\mathrm{Fe}/\mathrm{Cr}{\left[110\right]}^{n\mathrm{Cr}}$ in Fe-13Cr, and those of the vacancy and the [110] self-interstitial atom in α-Fe, and *n*(IS), and *n*(FS) are the nearest-neighbor Cr atoms around the defects in IS and FS states.

Type of Defect	Matrix	*n*(IS)	*n*(FS)	Labels	Barriers (eV)
V	α-Fe				0.74
[110]SIA	α-Fe				0.32
$V^{n\mathrm{Cr}}$	Fe-13Cr	1	1	1	0.57
		1	1	2	0.66
		1	1	3	0.55
		1	1	4	0.56
		1	2	-	0.43
		1	4	-	0.35
		1	8	-	0.60
		2	1	-	0.55
		4	1	-	0.48
		8	1	-	0.66
$\mathrm{Fe}/\mathrm{Cr}{\left[110\right]}^{n\mathrm{Cr}}$	Fe-13Cr	0	1	-	0.19
		1	0	-	0.09
		1	1	1	0.27
		1	1	2	0.22
		1	1	3	0.21
		1	1	4	0.31
		1	8	-	0.32
		2	4	-	0.45
		4	2	-	0.38
		8	1	-	0.49

## Data Availability

Data sharing is not applicable to this article.
